# Assessment of Two Years' Sputum Smear Results among Tuberculosis Suspected Patients in Gambella Regional Hospital, Western Ethiopia

**DOI:** 10.1155/2018/1306075

**Published:** 2018-02-06

**Authors:** Mistire Wolde, Alemayehu Fereja

**Affiliations:** ^1^Department of Medical Laboratory Sciences, Addis Ababa University, Addis Ababa, Ethiopia; ^2^Gambella Regional Laboratory, Gambella, Ethiopia

## Abstract

**Background:**

Determining burden of pulmonary tuberculosis (PTb) may be associated with success of directly observed theraphy (DOT) services in high PTb areas, such as Gambella, Ethiopia.

**Objective:**

To assess burden of sputum smear positive SSP-PTb reports in Gambella Regional Hospital, in two consecutive years: mid 2013 to 2015.

**Methods:**

From March 2013 to February 2015, PTb suspected patients' sputum Acid Fast Bacilli test results were collected from laboratory registration book of Gambella Regional Hospital, western Ethiopia.

**Results:**

Of the total 3064 results, the proportions of males and individuals whose age is between 16 and 25 years were 1823 (59.5%) and 1046 (34.1%), respectively. Majority of samples were coming from newly PTb suspected patients (2587, or 84.4%); and of them, SSP-PTb cases were 9.9%. The overall SSP-PTb cases decreased by 2.1% in March 2014 to February 2015 as compared to March 2013 to Feburary 2014. Meanwhile, AFB smear negative cases were significantly associated with being male [1.384 (1.064–1.801)] and being of age group 16–25 years [1.517 (1.007–2.287)].

**Conclusion:**

In Gambella region, although the distribution of AFB smear results had no significant yearly variations and the overall burden of SSP cases was decreased, still a lot of work needs to be undertaken on the PTb prevention and control program.

## 1. Background

Tuberculosis (Tb) is major public health infectious disease, mainly caused by* Mycobacterium tuberculosis* (MTb) bacteria. It is the second leading cause of morbidity and mortality among infectious diseases. According to World Health Organization, WHO, 2011 report, currently around 9 million people are expected to acquire the disease. Ethiopia, a country located in Sub-Sahara Africa with the highest Tb prevalence recorded, is one of the 22 high Tb burden countries, with an estimated annual Tb incidence of 300/100,000 people and with prevalence and mortality rates of 480/100,000 and 54/100,000 people, respectively [1–3].

Acid Fast Bacilli (AFB) staining technique is one of the MTb diagnostic approaches and employed as a primary method in the examination of the bacilli from the given sample and also recommended by WHO as one of the strategic tools in the management of tuberculosis worldwide [3]. Demonstrating the AFB using Ziehl–Neelsen staining methods is the most affordable method in resource-poor countries including Ethiopia [4].

The aim of the current study was to assess burden of sputum smear positive results as measured by AFB smears in Gambella (western Ethiopia) Regional Hospital in the last two consecutive years (mid 2013 to 2015). Since report showed that Gambella Regional States have one of the high numbers of Tb cases detection in the country [5], assessing burden of AFB positives in the region may indirectly reflect the success of Tb control strategies in the region and in Ethiopia too.

## 2. Methods

**Figure 1:**
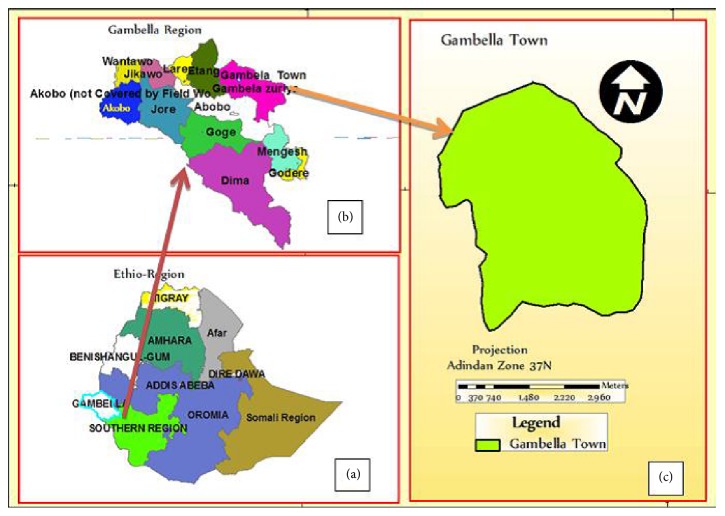
Gambella Regional State, Central Statistics Agency, Ethiopia. [7].

A retrospective study design was conducted to determine the trends of pulmonary tuberculosis among* pulmonary tuberculosis *suspected patients registered at Gambella Regional Hospital, western Ethiopia, from March 2013 to February 2015. Gambella is located at 777 KM west from Addis Ababa (capital city of Ethiopia), with a total population of 396,000 according to population projection 2014, by the Federal Democratic Republic of Ethiopia, Central statistical Agency [6]. Gambella Regional State has four administrative zones with eight districts and seven towns with a total of 174 kebeles (subcities), where eight of the kebeles are found in the capital city (Figure 1).

The Gambella Regional Hospital is one of the biggest hospitals in the western part of Ethiopia, assumed to deliver different healthcare services for up to 44, 269 people per year. Of the expected tasks by the hospital, provision of directly observed therapies (DOTs) for short course, for tuberculosis diagnosed patients, is the major one [7].

Although LJ culture tests are the golden standard in the diagnosis of MTb, the AFB smear results are also well recognized and highly recommended by WHO [3] on the overall management of MTb infection. Thus, according to our study protocol data was collected only from Gambella Hospital laboratory log book. All legible and fully documented data were included, while illegible and missed information's records were excluded from data collection. Consequently, convenient data collection took place by one of the authors, by considering all laboratory result registration log books for the past two years. The other author supervised and cross-checked the validity of the data. Out of the total reviewed documents, according to our exclusion criteria, 12 datasets were excluded. On the structured data collection format, information regarding study participants' age, sex, whether tuberculosis suspects are new or follow-up cases, AFB test results, date, and months and years of diagnosis was gathered. Quality assurance of the collected data was made by checking completeness and consistency immediately after data collection. The collected data was summarized on the same day of the data collection.

## 3. Data Analysis

Data was entered and analyzed by using Statistical Package for Social Sciences (SPSS) version 20 statistical software. Frequency, chi squares, and logistic regressions (both bivariable and multivariable) methods were employed. The analyzed variables were statistically significant, when *P* value was less than 0.05. Final results of analysis were displayed by tables and figures. Permission was obtained from Gambella Regional Hospital.

## 4. Ethical Clearance

All the study participants' samples were collected and processed, and final results were confidentially kept at the Gambella Regional Hospital. Therefore, to access these data from laboratory log book and conduct the study, permission was obtained from Gambella Regional State, Health Bureau, with reference number 14/7318/2 on 10/10/2015. Confidentiality of data was maintained throughout the study by keeping hard copies locked and electronic files' password protected.

## 5. Results

A total of 3064 sputum examination results were reported for tuberculosis suspected patients during the study period. Most of patients were males (59.5%), 16–35 years of age (61.8%), new (84.4%), and tested from March 2013 to February 2014 (51.3%) (Table 1).

The proportion of AFB sputum smear positive results was reduced by 2.1% in the last study period, March 2014–Feb. 2015, as compared to previous year, March 2013–February 2014 (Table 2). Of the total 2587 newly suspected pulmonary tuberculosis patients, the overall prevalence of AFB positive cases was 9.9% (256/2587). Moreover there was significant difference between males and females tuberculosis patients, as well with different age groups, *P* < 0.05; meanwhile, there was no significant difference between the numbers of examined sputum samples during the two years' period as shown in Table 2.

On the monthly distribution of PTb suspected cases in Gambella Hospital, AFB sputum smear positive cases were relatively high during the months of January, March, April, May, September, October, and November in the year between March 2013 and Feb. 2014 (Table 3), while in the time period between March 2014 and February 2015 smear positive pulmonary tuberculosis cases were relatively high in the month of January, March, May, June, July, and August (Table 3). But there was no significant difference in terms of number of PTb suspects and AFB sputum smear positive cases between similar months' results in the two consecutive years.

On multinomial logistic regression analysis, AFB smear negative results were significantly associated with male study participants compared to females [1.384 (1.064–1.801)], and these values were not significantly changed after adjusting for different age groups, year, and different monthly distribution of AFB test results with odds ratios of 1.363 (1.046–1.7750) (Table 4). Similarly, the AFB smear negative results were significantly associated with age group between 16 and 25 years with odds ratios of 1.517 (1.007–2.287) as compared to other age groups. The association of AFB smear negative results with age groups between 16 and 25 years as compared with other age categories did not significantly change even after being adjusted for sex, years, and different monthly distribution of AFB test results, with odds ratios of 1.532 (1.015–2.313) (Table 4). Since there was no statistically significant difference between years (Table 2), year of diagnosis was not considered in the logistic regression model.

## 6. Discussion

In the present study, the majority of pulmonary tuberculosis suspects were male accounting for 1823 (59.5%). Similar findings were also seen in the nearby town Mizan Aman, southwestern Ethiopia [8], and Harer, eastern Ethiopia [9], where male participants were 57.22% and 56.5%, respectively [8, 9]. This might be related to more males having tendency to visit health institutions when they do have health problem or have more close social contact and gatherings to acquire the disease than females. Nevertheless in our finding, occurrence of relatively high AFB smear negative results in male study participants is different from what was done in other areas [8–11]. This might be associated with the number of male study participants being so high in our cases with negative results, and actually the AFB smear positive tests results show decreases in tuberculosis cases in area. But still it needs further additional large scale studies.

On the other hand, the current study demonstrated that the AFB smear negative cases were significantly high in age group 16–25 years as compared with age categories 45 and above. Our findings were similar to the study conducted by Tadesse et al. [10], where more AFB positive cases are seen above age group above 45 years. Nevertheless it differs from studies done by Sintayehu et al. [8] and Mekonnen [11] in different parts of Ethiopia, where AFB smear positive cases are high in age groups below 45 years. This might be associated with sampling methods and tendency of tuberculosis suspected individuals opportunity to visit healthcare institutions, as well clinicians tendency to order AFB tests.

The burden of smear positive pulmonary tuberculosis cases in Gambella Regional Hospital 9.9% which was lower than the study done by Mekonnen (a study done at the eastern part of Ethiopia) was 21.6% [11], and in another similar study conducted in Gambella region (between 2003 and 2012), a prevalence was reported to be 31.6% [12]. The decrease in the burden of smear positive PTb in the region could be related to success in the Tb control strategy program, or there might be poor quality of AFB smears and detection or patients may not visit healthcare facilities even if they are more likely to be tuberculosis suspects. Since AFB method is the right hand in the DOT program on the prevention and control of Tb, further studies are needed to assess the quality of AFB techniques in the Gambella region and other factors.

In the analysis of seasonal distribution of tuberculosis in Gambella region, there was no significant difference in the scattering of pulmonary tuberculosis between the last two years as well as during each month. Nevertheless, relatively high AFB slides had been examined in January, May, and July to diagnose pulmonary tuberculosis cases. This observation was similar to study conducted in Iran [13], but to some extent different from the study conducted in America [14]. Different suggestions and hypotheses were forwarded for the seasonal variation in the occurrence of Tb, as occurrence of Tb is more in winter season than in summer [15], due to cold weather, deficiency in vitamin 6 production in association with reduction in sun light intensity [16], and people living in crowded environment. Some of the justification may not apply for the present study, since Gambella is one of the hottest regions in Ethiopia. Thus seasonal variations on AFB positivity might be related to large family size and gathering of people after work in entertainment places, such as local bars and various religious activities.

## 7. Conclusions

In Gambella region, the proportion of AFB sputum smear positive PTb cases reduced by 2.1% in 2014/15, compared to 2013/14. More men visited the laboratory for sputum AFB tests than women during the study period, and pulmonary tuberculosis cases have shown some seasonal variations. Large scale and longitudinal research is highly recommended to describe the real trend of pulmonary tuberculosis in Gambella Regional Hospital. Moreover, continuous advocacy and health information is also required to reduce further the burden of tuberculosis in the region in particular and in Ethiopia at large.

## Figures and Tables

**Table 1:** Total frequency of sex, age, and pulmonary tuberculosis suspects in Gambella Regional Hospital, May 2013–February 2015.

Variable	Number	Percent (%)
Sex		
Male	1823	59.5
Female	1241	40.5
Age		
15 years and below	273	8.9
16–25 years	1046	34.1
26–35 years	848	27.7
36–45 years	456	14.9
46 years and above	441	14.4
Suspect *M. tuberculosis* cases		
New	2587	84.4
Follow-up	477	15.6
Period		
March 2013–Feb. 2014	1572	51.3
March 2014–Feb. 2015	1492	48.7

**Table 2:** Sex and age distribution of new AFB positive cases in Gambella hospital laboratory, from May 2013 to February 2015.

Variable	Sputum AFB result		*P* value
	Positive 256 cases, *n* (%)	Negative 2331 cases, *n* (%)	
Sex			
Male	170 (11.2)	1345 (88.8)	0.007
Female	86 (8.0)	986 (92.0)	
Age			
15 years and below	10 (3.8)	253 (96.2)	0.001
16–25 years	103 (12.5)	720 (87.5)	
26–35 years	72 (10.4)	618 (89.6)	
36–45 years	44 (10.6)	371 (89.4)	
46 years and above	27 (6.8)	369 (93.2)	
Period			
March 2013–Feb. 2014	139 (11.0)	1130 (89.0)	0.077
March 2014–Feb. 2015	117 (8.9)	1201 (91.4)	

**Table 3:** Monthly distribution of AFB sputum smear results in Gambella Regional Hospital, from March 2013 to February 2014 and from March 2014 to February 2015.

Month	AFB sputum smear result	From March 2013 to February 2014^#^	From March 2014 to February 2015^*∗*^
January	% of AFB+	10.8	13.7
	% of AFB−	8.3	7.8
February	% of AFB+	7.9	7.7
	% of AFB−	10.9	11.2
March	% of AFB+	7.9	8.5
	% of AFB−	6.8	8.1
April	% of AFB+	9.4	6.0
	% of AFB−	7.9	8.6
May	% of AFB+	10.1	11.1
	% of AFB−	8.8	8.7
June	% of AFB+	7.9	9.4
	% of AFB−	8.4	7.1
July	% of AFB+	10.8	12.0
	% of AFB−	15.0	10.6
August	% of AFB+	2.9	8.5
	% of AFB−	6.3	6.2
September	% of AFB+	7.9	6.8
	% of AFB−	6.5	7.0
October	% of AFB+	9.4	3.4
	% of AFB−	6.1	6.6
November	% of AFB+	9.4	6.8
	% of AFB−	6.4	9.2
December	% of AFB+	5.8	6.0
	% of AFB−	8.6	8.8

^#^From March 2013 to Feb. 2014, total AFB sputum stain positives and negatives were 139 and 1130, respectively. ^*∗*^From March 2014 to Feb. 2015, total AFB sputum smear positives and negatives were 117 and 1201, respectively.

**Table 4:** Logistic regression of AFB smear negative cases versus age and sex distribution in Gambella Hospital from March 2013 to February 2015.

Variable	AFB test results	
	COR with 95% CI	AOR with 95% CI^#^
Sex		
Male	1.384 (1.064–1.801)^*∗*^	1.363 (1.046–1.775)^*∗*^
Female	1	1
Age		
15 years and below	0.486 (.235–1.005)	0.505 (0.244–1.046)
16–25 years	1.517 (1.007–2.287)^*∗*^	1.532 (1.015–2.313)^*∗*^
26–35 years	1.204 (0.781–1.855)	1.217 (0.789–1.877)
36–45 years	1.399 (0.872–2.247)	1.402 (0.873–2.253)
46 years and above	1	1

^#^Adjusted for sex, age, and year and monthly distribution of AFB test results; ^*∗*^*P* < 0.005.

## Abbreviations

AFB: Acid Fast Bacilli
DOT: Directly observed therapy
MTb: * Mycobacterium tuberculosis*
PTb: Pulmonary tuberculosis
SPSS: Statistical Package for Social Sciences
Tb: Tuberculosis
WHO: World Health Organization.

## Additional Points

*Limitations of the Study.* This paper had limitations regarding collection of the exact addresses of study participants in Gambella regions and progress of Tb treatments after diagnosis as well as information on humidity and rainfall to explain seasonal variations of Tb. Moreover, the diagnosis of pulmonary tuberculosis entirely depends on only smear status which has low sensitivity compared to culture. If we have used culture, the rate of pulmonary tuberculosis would significantly increase. It does not necessarily show burden of tuberculosis in the two years' time.
